# Impact on mortality of being seropositive for hepatitis C virus antibodies among blood donors in Brazil: A twenty-year study

**DOI:** 10.1371/journal.pone.0226566

**Published:** 2019-12-19

**Authors:** Hélio Ranes de Menezes Filho, Ana Luiza de Souza Bierrenbach, Maria Ligia Damato Capuani, Alfredo Mendrone, Adele Schwartz Benzaken, Soraia Mafra Machado, Marielena Vogel Saivish, Ester Cerdeira Sabino, Steven Sol Witkin, Maria Cássia Mendes-Corrêa

**Affiliations:** 1 Department of Infectious Diseases, University of São Paulo, School of Medicine, São Paulo, SP, Brazil; 2 Department of Health Sciences, Federal University of Jataí, Jataí, GO, Brazil; 3 Instituto de Ensino e Pesquisa, Hospital Sírio-Libanês, São Paulo, SP, Brazil; 4 Hemocentro de São Paulo, Fundação Pró-Sangue, São Paulo, SP, Brazil; 5 Heitor Vieira Dourado, Tropical Medicine Foundation, Manaus, Amazon, Brazil; 6 Department of Obstetrics and Gynecology, Weill Cornell Medicine, New York, New York, United States of America; 7 Institute of Tropical Medicine, University of São Paulo, São Paulo, SP, Brazil; Centers for Disease Control and Prevention, UNITED STATES

## Abstract

**Introduction:**

Hepatitis C virus (HCV) infection is a major health problem associated with considerable risk of mortality in different regions of the world. The purpose of this study was to investigate the contribution of HCV infection on all-cause and liver-related mortality, in a large cohort of blood donors in Brazil.

**Methods:**

This is a retrospective cohort study of blood donors from 1994 to 2013, at *Fundação Pró-Sangue—Hemocentro de São Paulo (FPS)*. This cohort included 2,892 and 5,784 HCV antibody seropositive and seronegative donors, respectively. Records from the FPS database and the Mortality Information System (SIM: a national database in Brazil) were linked through a probabilistic record linkage (RL). Mortality outcomes were defined based on ICD-10 (10th International Statistical Classification of Diseases and Related Health Problems) codes listed as the cause of death on the death certificate. Hazard ratios (HRs) were estimated for outcomes using Cox multiple regression models.

**Results:**

When all causes of death were considered, RL identified 209 deaths (7.2%) among seropositive blood donors and 190 (3.3%) among seronegative blood donors. Donors seropositive for HCV infection had a 2.5 times higher risk of death due to all causes (95% CI: 1.76–2.62; p<0.001). When only liver-related causes of death were considered, RL identified 73 deaths among seropositive blood donors and only 6 among seronegative blood donors. Donors seropositive for HCV infection had a 23.4 times higher risk of death due to liver related causes (95% CI: 10.2–53.9; p<0.001). Donors seropositive for HCV had a 29.5 (95%CI: 3.9–221.7), 2.8 (95% CI: 1.4–5.5) and a 1.9 (95% CI: 1.2–3.0) times higher risk of death due to hepatocellular carcinoma, infection or trauma, respectively, compared to seronegative donors.

**Conclusions:**

All-cause and liver-related mortality rate was increased among blood donors seropositive for HCV compared with the mortality rate among seronegative blood donors. Our data confirms HCV as a relevant cause of death in Brazil and also suggest that interventions directed at following patients even after access to specific drug treatment are urgent and necessary.

## Introduction

Hepatitis C virus (HCV) infection is a major health problem associated with considerable risk of mortality in Europe, United States, Canada and Australia [1–7]. In all these areas, mortality rates are higher among individuals positive for HCV antibodies than for the general population [1–7]. This infection affects about 71 million people worldwide, accounting for about 400,000 deaths per year [8].

HCV is a hepatotropic virus that causes a slowly progressive liver disease leading to the development of cirrhosis in approximately 10–20% of infected individuals over a 20–30 year period [9]. It is the leading cause of end-stage liver disease, hepatocellular carcinoma (HCC) and liver-related death in many parts of the world [9]. In addition to manifestations associated with the evolution of liver disease, HCV infection is associated with numerous extrahepatic complications, which may also impact survival. It is estimated that 40–74% of patients with HCC may develop at least one extrahepatic manifestation during the course of the disease [10].

In Brazil, it is estimated that about 700,000 people have a chronic HCV infection. In recent years, the Minister of Health has made efforts and investments to implement a strategy aimed at eliminating HCV infection in this country, notably increased access to diagnostic tests and treatment with direct-acting antivirals. The Brazilian public unified health system (SUS) provides free-of-charge and universal treatment for all HCV-infected patients. From 2014 until December 2017, 70,000 people received treatment with DAA at an approximate cost of US$1billion [11, 12]. An evaluation of the mortality rates associated with HCV infection in Brazil as well as a delineation of the causes of death in this population may improve the focus of intervention strategies to reduce the consequences of chronic HCV infection, which include progressive liver disease and other extra-hepatic illnesses. Also, as the maintenance of some of the Brazilian SUS programs is currently being revised by a new political administration, this kind of information, i.e., the clear delineation of HCV differential mortality risks, may be relevant to guide public health policy and to guaranteeing the continuation of the viral hepatitis programs.

Having the possibility to follow-up a cohort of blood donors with known serological status for many infectious diseases is a major study opportunity, as they go through an interview before donating and requirements to donate include being in good health. We therefore believe that donors may be a convenient sample with a similar profile to the local population of the same age-range.

We therefore performed a retrospective cohort study of otherwise healthy blood donors screened for HCV antibodies at blood donation. The purpose of this study was to investigate the contribution of HCV infection on all-cause and liver-related mortality as well as identifying the main underlying causes of death in a large cohort of blood donors in Brazil.

## Methods

### Study design

This retrospective cohort study compares mortality rates and causes of death among blood donors who were seropositive or seronegative for HCV over a maximum period of 22 years.

The study period was 1994–2016. Study subjects donated blood between 1994 and 2013 at *Fundação Pró-Sangue—Hemocentro de São Paulo (FPS)*, one of the largest blood banks in Brazil and located in the capital city of São Paulo State [13]. Donors entered the cohort on the date of their donation and were monitored over time until their deaths or, if they survived, until December 31, 2016, which was the last available date of death in the Mortality Information System (SIM) database. Records from the FPS database and the SIM were linked to ascertain whether donors had died in the period between 2000 and 2016.

### Data sources

The study used the two aforementioned individual-based databases with nominal information, FPS and SIM. The FPS database contained nominal information on all donors, including name, mother’s name, date of birth and address. It also contained the following variables: serological test results, sex, age, date of donation, whether this was a first-time donor, and whether the donor was voluntary or a replacement donor. A voluntary or spontaneous donation is one in which the donor presents himself to donate by his own free choice, while the replacement donation aims to replace the blood that has already been used or when it has the name of a possible recipient. Specific donation occurs when a person donates to a particular recipient, and autologous donation, in turn, is when someone donates to oneself [13].

The SIM database supports the collection, storage and management process of death registries in Brazil, which is mandatory in all cities. The Ministry of Health in Brazil manages the system. Data are based on the international form of the death certificate. Causes of death (COD) are usually assigned by the attending physicians or, if needed, by pathologists in the Death Investigating Services (SVOs) or coroners from the Forensic Institutes (IMLs). Coding of COD into the International Classification of Diseases, 10th Edition (ICD-10) codes is undertaken using an automated coding system and reviewed by certified coders [14, 15]. The SIM database that was available for the study contained the following variables: name, mother’s name, date of birth, address, sex, age, date of death, and CID-10 codes for the underlying and associated causes of death.

Mortality data coverage and quality (represented by a smaller proportion of COD with ill-defined/ garbage codes) have increased over the last 40 years, as shown in a recent international comparison. Even though improvements continue to happen, during our study period such indicators already had intermediate to high levels with smaller variation [16].

### Study population and exposure definitions

The population included in our study cohort consisted of all donors who tested seropositive for HCV between 1994 and 2013, plus a random sample of donors who were seronegative for all screening tests performed at the FPS at a ratio of approximately 2:1 and matched by year of blood donation. The minimum number of members for each of the groups was calculated using Stata software, version 14.0. A significance level of 95% (α = 0.05), a statistical power of 80% (β = 20%), an exposed / unexposed ratio of 1: 2 and a relative risk of 3.12 were considered in the mortality among exposed (HCV +) and unexposed (HCV-) individuals. Thus, the required sample size was 350 HCV + and 700 HCV- individuals. During the study period, the FPS routinely performed the following serology screening tests on all donations before releasing units for transfusion: HCV, hepatitis B virus (HBV: HBsAg and AntiHBc), syphilis, human immunodeficiency virus (HIV), human T-cell lymphotropic viruses I and II (HTLV), and Chagas disease [17].

During the study period (1994–2013) FPS screened all donors for HCV infection by enzyme-linked immunosorbent assay (ELISA) and RIBA HCV 3.0 strip immunoblot assay [18]. HCV-positive donors were defined as those who tested positive for EIA with RIBA HCV 3.0 or Western blot followed by a positive EIA [17, 19–21]. Nucleic acid amplification tests (NAAT) for HCV diagnosis were not required in Brazil until November 2013. The diagnostic tests used for sample screening had different sensitivity and specificity values. First-generation ELISAs had a sensitivity of 92% in hemophiliacs and 84% in hepatitis non A non B patients. Second generation ELISAs had 100% sensitivity in both cases [22]. Regarding the third generation ELISA, they presented sensitivity of 97.2% (95% confidence interval: 92–99%) in patients with chronic liver disease, 100% in hemodialysis patients and 98.9% (CI: 94–100%) in serum panels. Specificity was 100% in hemodialysis patient samples and in patients with chronic liver disease [23]. Fourth generation ELISA had the advantage of simultaneous detection of antigen and viral antibody, with sensitivity of 70.5% of HCV RNA positive and anti-HCV negative samples collected in the pre-conversion phase. Specificity was estimated at 99.8% (95% confidence interval [CI], 99.6%-99.97%) [24]. The RIBA 3.0 test (Chiron Corporation, Emeryville, USA) was evaluated for sensitivity and specificity considering HCV RNA detection as the gold standard: both were 100% in patients with chronic liver disease and 78.8% (CI: 65–89%) and 80% (CI: 30–96%), respectively, in hemodialysis patients [23]. NAT has a sensitivity of 1,800 IU / ml (minipool of six samples—MP6) and 300 IU / ml in the individual samples [25]. Those HCV-positive donors who were also positive for one or more of the other infections tested were considered to be co-infected. In our primary analyses, we compared mortality outcomes of HCV-seropositive donors to those of donors who were seronegative for all other screening tests. Further details on model specifications are provided below.

### Record linkage procedures

To identify blood donors who had died during the follow-up period, record linkage (RL) was performed to link records from the FPS and SIM databases. Due to the absence of a national identity number, a two-step RL procedure was used. First, an in-house linkage algorithm was developed by staff members from the Brazilian Ministry of Health in which the probability that the two records would belong to the same patient was based on the person’s name and date of birth. A Bloom filter, which is a type of space-efficient probabilistic data structure, was constructed for each record following the methods developed by Schnell et al [26]. Because high sensitivity was achieved in this step at the expense of low specificity, a second step was necessary to increase the specificity of the matches found in the first step. The second step was performed by the study researchers using the Reclink III open source software [27]. Patients’ name, mother’s name, and date of birth were chosen as the matching variables. Three blocking step strategies were used based on combination of the phonetic codes of the variables first name, last name and gender. The first blocking step used gender and the phonetic codes of the first name and last name. The second blocking step used only gender and the phonetic code of the first name, and the third blocking step used only gender and the phonetic code of the last name. The RL procedure used has been validated in a previous publication, in which linkage results were compared from blood donors whose vital status was known [28]. The results of this study indicated a sensitivity of 94% (95%CI, 90–97%) and a specificity of 100% (95%CI, 98%-100%).

Unfortunately, the SIM database contained personal information only beginning in 2000, so the researchers could not perform RL between FPS and SIM for earlier time periods. For the cohort that donated blood in 1994, we were unable to consider the first six years of follow up. For the 1995 cohort, we were unable to consider the first five years of follow up. In other words, regardless of the patients’ HCV serology status, we do not know whether those who donated blood in 1994, for example, died in the period between 1994 and 1999. However, based on findings of seropositive and seronegative individuals who donated blood in 1999 and from whom we lacked data only on deaths that may have occurred during the year of their blood donation, we found that deaths were extremely rare in the first year after donation. Because of this finding and in an effort to work around this limitation to the data, we made the analytical assumption that no one in our cohort died between 1994 and 1999. In order to check if this assumption could indeed be made, we also performed a sensitivity analysis in which we repeated the logistic regression models restricting the years included in our cohorts.

### Mortality outcomes

Donors entered the cohort on the date of their donation and were monitored over time until their deaths or, if they survived, until December 31, 2016, which was the last available date of death in the SIM database.

Thus, in the first analysis, we compared deaths between hepatitis C mono-infected donors and seronegative donors for all diseases. Hepatitis C mono-infected donors were defined as blood donors found to be seropositive only for hepatitis C virus infection. Seronegative donors were defined as blood donors negative for all tested infections, as detailed above: hepatitis C virus, hepatitis B virus, syphilis, HIV, HTLV and Chagas disease.

For this analysis, mortality outcomes were defined based on the ICD-10 codes listed as the underlying cause or any of the associated causes of death on the main portion of the death certificate [29]. The underlying cause of death was understood to mean the disease or injury that initiated the chain of pathological events that led directly to death, or the circumstances of the accident or violence that produced the fatal injury [14,15]. In this first analysis, the two groups were compared for all-cause mortality and for mortality associated or directly related to HCV. In this context, a group of experts suggested a list of ICD-10 that could be considered as causes of mortality associated or directly related to HCV: B17.1 (Acute hepatitis C), B18.2 (Chronic viral hepatitis C), B18.8 (Other chronic viral hepatitis), B18.9 (Chronic viral hepatitis, unspecified), B19.0 (Unspecified viral hepatitis with hepatic coma), B19 (Unspecified viral hepatitis), C22.0 (Liver cell carcinoma), C22.9 (Malignant neoplasm of Liver, unspecified), I85.0 (Esophageal varices with bleeding), K72.0 (Acute and subacute hepatic failure), K72.1 (Chronic hepatic failure), K72.9 (Hepatic failure, unspecified), K73.0 (Chronic persistent hepatitis, not elsewhere classified), K73.1 (Chronic lobular hepatitis, not elsewhere classified), K73.2 (Chronic active hepatitis, not elsewhere classified), K73.8 (Other chronic hepatitis, not elsewhere classified), K73.9 (Chronic hepatitis, unspecified), K74.0 (Hepatic fibrosis), K74.1 (Hepatic sclerosis), K74.2 (Hepatic fibrosis with hepatic sclerosis), K74.6 (Other and unspecified cirrhosis of liver), K75.9 (Inflammatory liver disease, unspecified), K76.6 (Portal hypertension), K76.9 (Liver disease, unspecified).

In a second analysis, to be able to compare our results with those of a similar study by Guiltinan et al [1] and in order to further qualify the comparison between seropositive and seronegative, we also defined a list of groups of causes of death based on codes listed exclusively as the underlying cause of death. We used the same grouping strategy as was identified in the prior study, with minor modifications (S1 Table): HCV, Other viral hepatitis, Liver Disease, Liver Cancer, Cancer—excluding liver, Cardiovascular, Pulmonary, Trauma, Suicide, Drug/alcohol, Neurological Diseases, Infection, Other/unknown—Renal diseases, Other/unknown—Diabetes mellitus, Other/unknown–Other.

### Statistical analysis

Categorical variables were compared using the Pearson’s Chi-square test or Fisher’s exact test. Mortality rates were calculated by dividing the number of deaths by the population at risk during the follow-up period, i.e. the person-years accrued in our cohort.

Hazard ratios (HRs) were estimated for outcomes using Cox multiple regression models. The covariates sex, age, status as a first time donor, and type of donor motivation (voluntary or replacement) were tested as potential confounders and effect modifiers in all models. Age was analyzed as a continuous variable, while the others were treated as binary variables. These were the only variables available for analyses in the FPS database. Backward selection procedures were used to determine which of the covariates should remain in the models. Comparing the adjusted and unadjusted HRs for each covariate assessed confounding. Likelihood ratio tests assessed the contribution of each variable and their modification effects on the models. The proportional hazards assumption was tested statistically on the basis of Schoenfeld residuals and by checking the parallelism of the curves in log-log plots. Consistently, results indicated no evidence of violation of the assumption. Kaplan-Meier curves were used to analyze the differences in survival associated with exposure variables and statistical differences tested with the Log-rank test. Given the multiple comparisons, the Bonferroni correction method was used to set the significance level to be used.

As mentioned previously, a sensitivity analysis was performed to compare risks of death by restricting the years included in our cohorts. In other words, we ran two sets of analyses: one considering all blood donors who donated from 1994 to 2013, and the other considering only those from 2000 to 2013. Statistical analyses were conducted using the Stata software, version 13.1 (StataCorp LP, College Station, TX, USA).

### Ethical approval

The study was approved by the Research Ethics Committee of Clinical Hospital of the University of Sao Paulo School of Medicine, or HC-FMUSP (CAAE Registry No.: 62572616.5.0000.0065). The committee deemed it unnecessary to obtain informed consent from each individual for the review of their medical records due to the difficulty of obtaining such consent and the retrospective nature of the two databases. All individual identifiers were removed from the database after the probabilistic RL was performed.

## Results

Between 1994 and 2013, 2,892 donors who were seropositive for HCV (HCV+) were identified from the FPS sample. They were matched by year of donation to 5,784 seronegatives donors who were not reactive to any of the other serological tests (Table 1). Of the 8,676 subjects, 5,832 (67.2%) were male and 2,844 (32.8%) were female. There was a predominance of individuals between 17 and 29 years of age (46.8%); the second most common age group was 30 to 39 years (27.4%). A total of 51.6% volunteered to donate as a social service, and the majority (63.6%) were donating for the first time.

**Table 1:** Demographic characteristics of the study population organized by HCV serological status.

		Seropositive		Seronegative		p-Value
		N = 2,892		N = 5,784		
		N	(%)	n	(%)	
**Sex**						< 0.001
	Male	1,878	(64.9)	3,954	(68.4)	
	Female	1,014	(35.1)	1,830	(31.6)	
**Age Range (in years)**						<0.0001
	17–29	1,181	(40.8)	2,880	(49.8)	
	30–39	709	(24.5)	1,670	(28.9)	
	40–49	687	(23.8)	894	(15.5)	
	>50	310	(10.7)	334	(5.8)	
	Unknown	5	(0.2)	4	(0.1)	
**Type of Donation**						<0.0001
	Voluntary	1,399	(48.4)	3,082	(53.3)	
	Replacement Donor	1,487	(51.4)	2,686	(46.4)	
	Specific	6	(0.2)	16	(0.3)	
	Autologous	0	(0.0)	0	(0.0)	
**Type of Donor**						<0.0001
	First-time donor	2,763	(95.5)	2,753	(47.6)	
	Repeat donor	129	(4.5)	3,031	(52.4)	

There were differences between seropositive and seronegative donors. Seronegative donors were younger, had a higher proportion of males and voluntary and repeated donors (Table 1).

In the analysis of the rates and causes of death, six individuals were excluded due to inconsistent data, four of whom were seropositive and two of whom were seronegative. An additional 236 donors were excluded from the seropositive group due to their reactivity to another microorganism in the serological screening tests. Thus, a total of 8,434 subjects remained, 2,652 of whom were seropositive and 5,782 of whom were seronegative.

Table 2, which refers to the first analysis mentioned in the topic "Mortality Outcomes", shows that, between 1994 and 2016, when all causes of death were considered, there were 399 deaths between the two groups, 209 of which were in HCV+ individuals and 190 were in HCV- individuals. Thus, the crude hazard ratio (HR) was 2.5 (95%CI: 2.06–3.05; p<0.001), and the adjusted HR was 2.15 (95%CI: 1.8–2.6; p<0.001). As for causes of death related or associated to HCV, there were 73 deaths in the HCV+ group (mortality rate of 168.96 deaths per 100,000 person-years) and 6 deaths in the HCV negative group (mortality rate of 6.18 deaths per 100,000 person-years). Thus, the crude HR was 27.6 (95%CI: 12.0–63.4; p<0.001), and the adjusted HR was 23.4 (95%CI: 10.2–53.9; p<0.001).

**Table 2:** Deaths organized by cause of death and by HCV serological status.

Cause of Death	Number of Deaths		Mortality rate 100,000 PY*** (IC95%)		Crude HR^#^ (CI95%) p-Value	Adjusted ^&^HR^#^ (CI95%) p-Value
	Sero+* N = 2,652	Sero-** N = 5,782	Sero+ 43,206.366 PY	Sero- 97,047.702 PY		
Deaths due to all causes	209	190	483.73 (422.4–553.96)	195.78 (169.83–225.69)	2.50 (2.06–3.05) <0.001	2.15 (1.76–2,62) <0.001
Deaths by causes associated with or directly related to HCV	73	6	168.96 (134.32–212.52)	6.18 (2.78–13.76)	27.55 (11.98–63.35) <0.001	23.41 (10.15–53.94) <0.001

*Sero+: HCV seropositive only

***PY: Person-years

**Sero-: Seronegative for all infections tested

^#^HR: Hazard Ratio

^&^HR adjusted for sex, age. Type of donation and type of donor dropped from final adjusted models. Male sex and increasing age acted as positive confounders on both adjusted models.

In the sensitivity analysis performed to compare risks of death restricting the years included in our cohorts to only those from 2000 to 2016, results obtained were equivalent to those observed on Table 2 (S2 Table).

**Fig 1:**
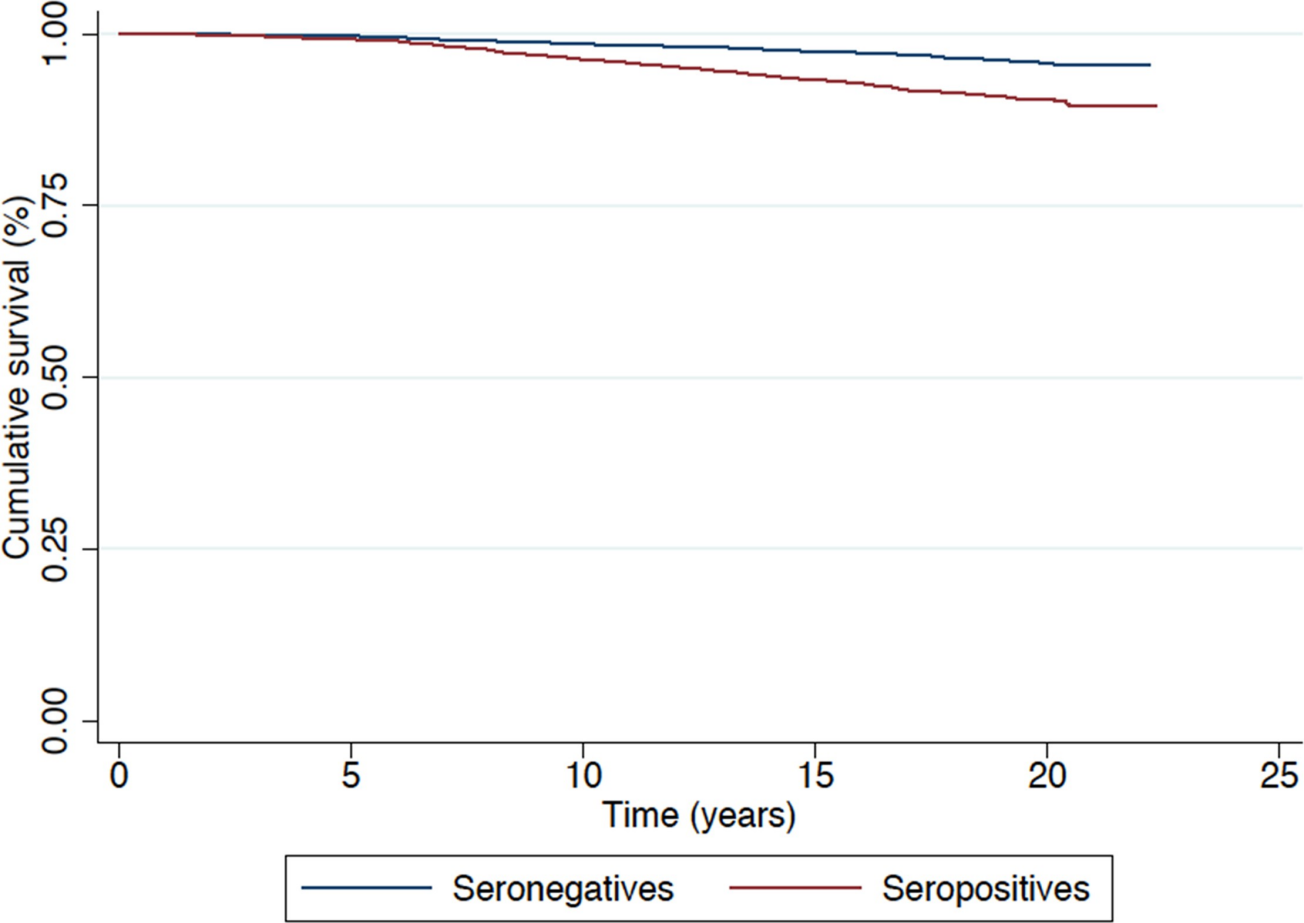
Survival (in years) by HCV serological status considering all causes of death.

**Fig 2:**
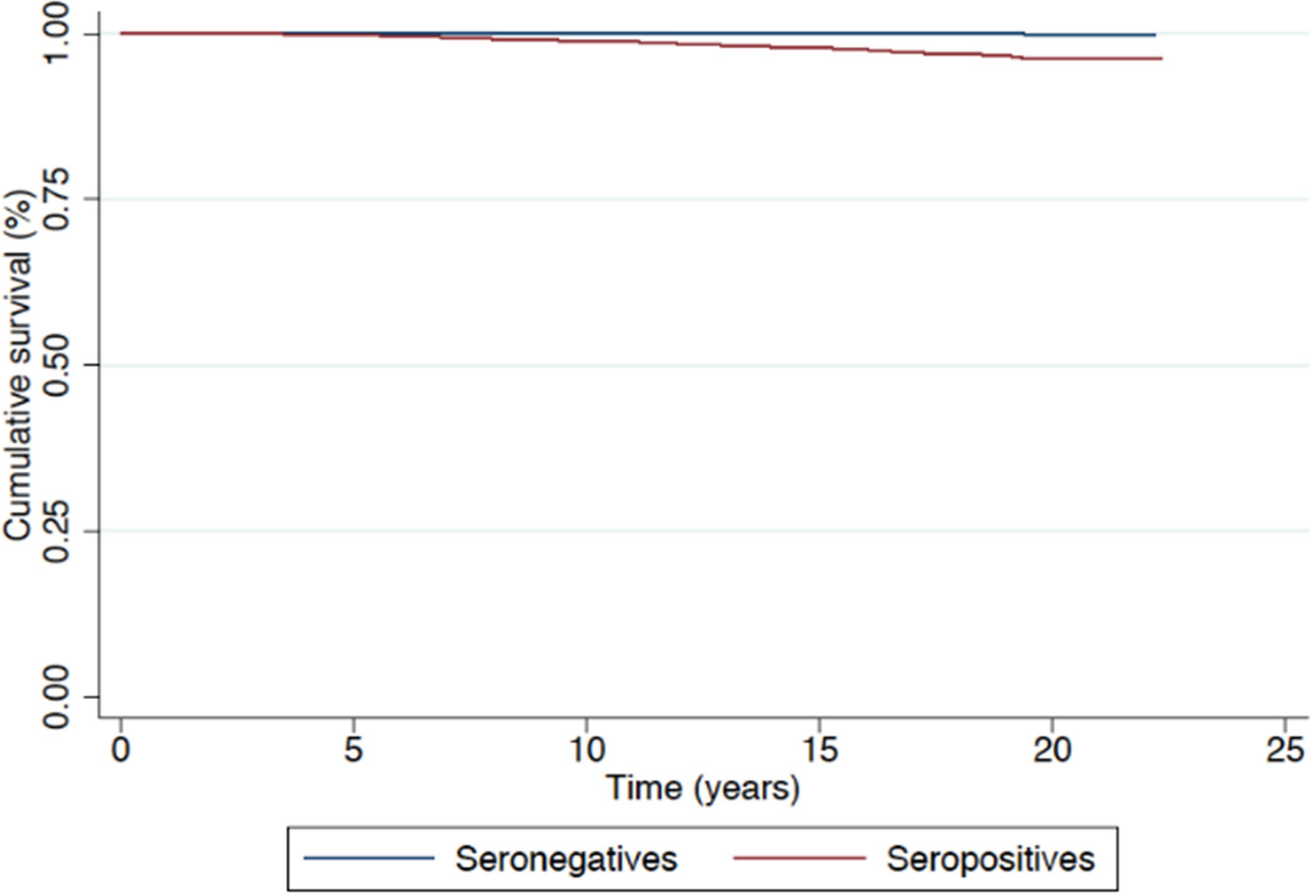
Survival (in years) by HCV serological status considering only causes of death related to HCV or associated with infection due to the virus.

Figs 1 and 2 show the survival curves based on cause of death. Higher rates of survival were found in the seronegative group for both groups of causes.

Table 3, which refers to the second analysis mentioned in the topic "Mortality Outcomes", provides data on the underlying causes of death, organized into categories. In addition to a predominance of deaths in the seropositive group, this group also differed significantly in terms of the following causes: liver related causes (HR = 24.1; 95%CI: 10.5–55.4), particularly of liver disease / cirrhosis (HR = 15.2; 95%CI: 5.3–43.4) and of liver cancer (HR = 29.5, 95%CI: 3.9–221.7); trauma (HR: 1.9; 95%CI: 1.2–3.0); and infection (HR = 2.8, 95%CI: 1.4–5.5).

**Table 3:** Deaths organized by underlying cause of death and by HCV serological status.

General Cause of Death	HCV+*	HCV-**	HR adjusted^#^	CI 95%	p-Value
	N = 2,652	N = 5,782			
**All liver related deaths**	75	6	24.06	(10.45–55.39)	**<0.001**
HCV	23	0	∞	-	-
Other viral hepatitis infection	4	1	7.10	(0.78–64.52)	0.082
Liver Disease	30	4	15.22	(5.34–43.37)	<0.001
Liver Cancer	18	1	29.52	(3.93–221.73)	0.001
**Non-Hepatic Cancer**	**28**	**48**	**1.00**	**(0.62–1.60)**	**0.992**
**Cardiovascular-Related Causes**	**17**	**29**	**1.03**	**(0.56–1.88)**	**0.931**
**Pulmonary Causes**	**9**	**8**	**2.32**	**(0.89–6.06)**	**0.086**
**Trauma/Suicide**	**32**	**45**	**1.66**	**(1.05–2.61)**	**0.029**
Trauma	32	40	1.86	(1.17–2.97)	0.009
Suicide	0	5	∞	-	-
**Drugs/Alcohol**	**2**	**0**	**∞**	**-**	**-**
**Neurological Diseases**	**7**	**13**	**0.90**	**(0.36–2.28)**	**0.830**
**Infection**	**20**	**14**	**2.75**	**(1.38–5.47)**	**0.004**
**Other/Unknown**	**19**	**27**	**1.39**	**(0.77–2.52)**	**0.277**
Kidney Diseases	3	4	1.26	(0.28–5.72)	0.761
Diabetes Mellitus	1	5	0.33	(0.38–2.87)	0.315
Other	15	18	1.74	(0.87–3.47)	0.117
**All Deaths**	**209**	**190**	**2.15**	**(1.76–2.62)**	**<0.001**

* HCV +: HCV seropositive only.

** HCV-: Seronegative for all infections tested.

#HR: HazardRatio adjusted by sex, age. Type of donation and type of donor dropped from final adjusted models. Male sex and increasing age acted as positive confounders on both adjusted models. CI: Confidence Interval

## Discussion

Data from this retrospective cohort study show that the mortality rate for liver-related or non-liver related causes was higher over a 22-year period of observation among former blood donors in Brazil who were seropositive for HCV as compared to seronegative blood donors. Liver disease and hepatocarcinoma were associated with a fifteenfold and a thirtyfold increase respectively, in overall mortality among HCV-positive blood donors. In addition, trauma and infection as primary causes of death were associated with twofold and threefold increases respectively, in overall mortality in HCV-positive individuals. These results are in accordance with prior studies in different settings and populations, demonstrating that HCV infection increases the risk of death when compared to individuals not infected with this virus [1–7].

Liver-related deaths are the most common cause of death among HCV-infected individuals [1–7]. However, in groups where intravenous drug use is the likely mechanism of HCV transmission, other causes of death may prevail. It has been suggested that mortality associated with HCV can be divided into two phases. In the first phase, death occurs most frequently in young people and is closely associated with a drug-using life style; in the second phase it is mostly related to chronic HCV-induced liver disease [7].

The mechanisms of HCV transmission appear to have changed over the last few years in Brazil. Prior to the initiation of blood screening in Brazil in 1992, blood and blood product transfusion were the predominant route of HCV transmission [30]. Injection drug use was also an important mode of transmission [30, 31]. Since that time, new screening protocols have rendered the blood supply safe [32]. In addition, a significant reduction in the overall frequencies of drug injection and needle-sharing has been observed, which has contributed to a reduction in hepatitis C transmission and the number of viremic cases [33–35]. Nowadays, nosocomial transmission (particularly hemodialysis) as well as transmission through needle sharing in nonmedical settings seem to account for the majority of new hepatitis C cases reported [36, 37]. Nevertheless, the number of cases in Brazil of compensated (325,900) and decompensated (45,000) cirrhosis, HCC (19,100), and liver-related deaths (16,700) is still expected to increase and will peak between 2028 and 2032, according to a recent mathematical modeling study [11].

An association between hepatitis C virus infection and neuropsychiatric symptoms, mainly depression, has been debated for some years [35]. A recent meta-analysis has indicated that depression is the most prevalent HCV-associated extra-hepatic manifestation (24.5% in HCV-positive vs. 17.2% in HCV-negative individuals) [35]. Major depressive disorders have been associated with an increased risk of unintentional injuries and different types of trauma [18]. The increase in mortality associated with trauma in HCV-positive individuals observed in our study could be associated to a variable extent with a higher rate of risk behaviors among former illicit intravenous drug users and or people with psychiatric disorders.

In our study, infections were associated with almost a threefold increased risk of death in HCV-positive individuals. An association between HCV infection and an increased risk of bacterial infections has previously been reported [38–42]. Tuberculosis, community-acquired pneumonia or bacterial infections among hemodialysis patients are much more frequent in HCV-infected individuals [38–42]. Co-infection with HIV and illicit intravenous drug use are also more common in HCV-infected patients and can lead to higher mortality rates from sepsis [43].

Bacterial infections among cirrhotic patients are also common, particularly in decompensated patients, and account for significant mortality [44]. Liver dysfunction leads to compromised humoral and cell-mediated immunity, which results in a decreased ability to protect the host from bacterial and other infections [44, 45].

Limitations of our study must be recognized. We were not able to determine the length of HCV infection in HCV-positive blood donors, which individuals were exposed to HCV but remained asymptomatic, which individuals cleared the virus after treatment or those who spontaneously eliminated the virus. In addition, we did not have data on other health risk behaviours, like tobacco or alcohol use or the presence of other co-morbidities, like diabetes mellitus and obesity, not only at the baseline time-point but also, throughout the study period for both groups.

Also, it is possible that some causes of death were not correctly reported. Improper filing of death certificates may have generated inadequate or misleading information on causes of death. However, such exposures were most likely comparable in both groups analyzed and thus, although additional studies are needed for verification, the effect of this methodological limitation on our findings most likely has been minimal. Additionally, use of the SIM database is considered the most appropriate and available method for assessing causes of death in Brazil.

We also had no information on the socioeconomic status of subjects in our cohort. Because lower socioeconomic status is associated with higher mortality rates, its omission may have biased to an unknown extent the observed survival differences. It is also important to note that we do not know how many seronegative donors at the time of donation became seropositive for hepatitis C virus infection at a later time period.

An advantage of our study is that we analyzed a cohort of blood donors. Blood donors are generally healthy people, and at least at the time of donation they were not carriers of any other blood-borne disease. In addition, the follow-up time of this population was quite long, reaching up to 22 years. These factors support the validity of our analysis of mortality associated with HCV in this population. We propose that the absence of a mortality database for the years 1994 to 1999, rendering us unable to ascertain whether or not donors in our cohort died during this period, may have had only a limited effect on our results. Early deaths after blood donation are not frequent, as observed for those in our cohort who donated from 2000 onwards. Our sensitivity analysis restricting the years included in our cohorts to only those from 2000 to 2016 produced similar results to those of the whole cohort.

It is also important to recognize that the mortality rate associated with HCV infection may remain higher than in the general population, even after this infection may have been successfully treated. Late complications of chronic liver disease, such as hepatocellular carcinoma and hepatic decompensation, as well as suicides and traumas may persist as primary causes of death even in those who achieved a virological cure [46, 47].

The Ministry of Health has invested heavily in resources to address and eliminate HCV infection in Brazil. A large output of financial resources, mainly focused on the cost of direct-acting drugs, has been available in recent years [12]. However, we believe that the data presented in this study, revealing that overall mortality is higher in the HCV-infected population, points to a need for additional comprehensive actions in conjunction with the increased use of medications. In terms of public health, it is necessary to establish a strategy of continuous monitoring of the HCV-infected population even after obtaining virological cure. This may be focused mainly on patients already identified with advanced liver disease, those with significant comorbidities or individuals with a history of neuropsychiatric disorders. Continued medical and patient education about the risks of liver-related morbidity and mortality from HCV infection will also be necessary.

In conclusion, in this retrospective cohort, the mortality rate from all causes was increased among blood donors who were seropositive for HCV compared to seronegative blood donors. Among the causes of death identified in the HCV-positive group complications of advanced liver disease, infections and trauma were frequent. These data confirm that consequences of HCV infection are a recognizable cause of death in Brazil and suggest that follow-up interventions after the administration of specific drug treatment are necessary to optimize survival.

## Supporting information

S1 Table ICD-10 codes included in specified disease groups. (DOCX)

S2 Table Deaths organized by cause of death and by HCV serological status (2000–2016). (DOCX)
