# Supplementary material for: Impact on mortality of being seropositive for hepatitis C virus antibodies among blood donors in Brazil: A twenty-year study
Source: PLoS One. 2019 Dec 19;14(12):e0226566. doi: 10.1371/journal.pone.0226566 (PMC6922443; doi:10.1371/journal.pone.0226566)
Supplement: S1 Table — (DOCX) [file pone.0226566.s001.docx]

**S1 Table*.* ICD-10 codes included in specified disease groups**

| **HCV** | |
| --- | --- |
| B171 | Acute hepatitis C |
| B182 | Chronic viral hepatitis C |
| **Other viral hepatitis** | |
| B162 | Acute hepatitis B without delta-agent with hepatic coma |
| B180 | Chronic viral hepatitis B with delta-agent |
| B181 | Chronic viral hepatitis B without delta-agent |
| **Liver disease** | |
| K703 | Alcoholic cirrhosis of liver |
| K704 | Alcoholic hepatic failure |
| K709 | Alcoholic liver disease, unspecified |
| K729 | Hepatic failure, unspecified |
| K739 | Chronic hepatitis, unspecified |
| K746 | Other and unspecified cirrhosis of liver |
| K760 | Fatty (change of) liver, not elsewhere classified |
| K769 | Liver disease, unspecified |
| **Liver cancer** | |
| C220 | Liver cell carcinoma |
| C229 | Malignant neoplasm of liver, not specified as primary or secondary |
| **Cancer – excluding liver** | |
| C069 | Malignant neoplasm of mouth, unspecified |
| C159 | Malignant neoplasm of esophagus, unspecified |
| C169 | Malignant neoplasm of stomach, unspecified |
| C187 | Malignant neoplasm of sigmoid colon |
| C189 | Malignant neoplasm of colon, unspecified |
| C19 | Malignant neoplasm of rectosigmoid junction |
| C20 | Malignant neoplasm of rectum |
| C250 | Malignant neoplasm of head of pancreas |
| C257 | Malignant neoplasm of other parts of pancreas |
| C259 | Malignant neoplasm of pancreas, unspecified |
| C260 | Malignant neoplasm of intestinal tract, part unspecified |
| C329 | Malignant neoplasm of larynx, unspecified |
| C342 | Malignant neoplasm of middle lobe, bronchus or lung |
| C349 | Malignant neoplasm of unspecified part of bronchus or lung |
| C37 | Malignant neoplasm of thymus |
| C419 | Malignant neoplasm of bone and articular cartilage, unspecified |
| C439 | Malignant melanoma of skin, unspecified |
| C445 | Other and unspecified malignant neoplasm of skin of trunk |
| C509 | Malignant neoplasm of breast, unspecified |
| C539 | Malignant neoplasm of cervix uteri, unspecified |
| C541 | Malignant neoplasm of endometrium |
| C55 | Malignant neoplasm of uterus, part unspecified |
| C61 | Malignant neoplasm of prostate |
| C679 | Malignant neoplasm of bladder, unspecified |
| C710 | Malignant neoplasm of cerebrum, except lobes and ventricles |
| C719 | Malignant neoplasm of brain, unspecified |
| C728 | Overlapping lesion of brain and other parts of central nervous system |
| C729 | Malignant neoplasm of central nervous system, unspecified |
| C780 | Secondary malignant neoplasm of lung |
| C790 | Secondary malignant neoplasm of kidney and renal pelvis |
| C80 | Malignant neoplasm without specification of site |
| C839 | Diffuse non-Hodgkin's lymphoma, unspecified |
| C859 | Non-Hodgkin lymphoma, unspecified |
| C900 | Multiple myeloma |
| C910 | Acute lymphoblastic leukemia |
| C969 | Malignant neoplasm of lymphoid, hematopoietic and related tissue, unspecified |
| D069 | Carcinoma in situ of cervix uteri, unspecified |
| **Cardiovascular** | |
| I10 | Essential (primary) hypertension |
| I119 | Hypertensive heart disease without heart failure |
| I219 | Acute myocardial infarction, unspecified |
| I248 | Other forms of acute ischemic heart disease |
| I251 | Atherosclerotic heart disease of native coronary artery |
| I253 | Aneurysm of heart |
| I259 | Chronic ischemic heart disease, unspecified |
| I421 | Obstructive hypertrophic cardiomyopathy |
| I509 | Heart failure, unspecified |
| I519 | Heart disease, unspecified |
| I710 | Dissection of aorta [any part] |
| I711 | Thoracic aortic aneurysm, ruptured |
| I713 | Abdominal aortic aneurysm, ruptured |
| I714 | Abdominal aortic aneurysm, without rupture |
| I99 | Other and unspecified disorders of circulatory system |
| Q213 | Tetralogy of Fallot |
| **Pulmonary** | |
| I269 | Pulmonary embolism without acute cor pulmonale |
| J449 | Chronic obstructive pulmonary disease, unspecified |
| J459 | Other and unspecified asthma |
| J690 | Pneumonitis due to inhalation of food and vomit |
| J81 | Pulmonary edema |
| J841 | Other interstitial pulmonary diseases with fibrosis |
| J90 | Pleural effusion, not elsewhere classified |
| J961 | Chronic respiratory failure |
| W84 | Unspecified threat to breathing |
| **Trauma** | |
| V021 | Pedestrian injured in collision with two- or three-wheeled motor vehicle in traffic accident |
| V050 | Pedestrian injured in collision with railway train or railway vehicle in nontraffic accident |
| V093 | Pedestrian injured in unspecified traffic accident |
| V193 | Pedal cyclist (driver) (passenger) injured in unspecified nontraffic accident |
| V284 | Motorcycle driver injured in non collision transport accident in traffic accident |
| V299 | Motorcycle rider (driver) (passenger) injured in unspecified traffic accident |
| V499 | Car occupant (driver) (passenger) injured in unspecified traffic accident |
| V685 | Driver of heavy transport vehicle injured in non collision transport accident in traffic accident |
| V892 | Person injured in unspecified motor-vehicle accident, traffic |
| W070 | Fall from chair |
| W109 | Fall (on) (from) unspecified stairs and steps |
| W139 | Fall from, out of or through building, not otherwise specified |
| W190 | Unspecified fall: home |
| W199 | Unspecified fall: unspecified place |
| W209 | Struck by thrown, projected or falling object: unspecified place |
| W349 | Discharge from other and unspecified firearms: unspecified place |
| W698 | Accidental drowning and submersion while in natural water: other specified place |
| W748 | Unspecified cause of accidental drowning and submersion: other specified place |
| W749 | Unspecified cause of accidental drowning and submersion: unspecified place |
| W879 | Exposure to unspecified electric current: unspecified place |
| X950 | Assault by other and unspecified firearm discharge: home |
| X954 | Assault by other and unspecified firearm discharge: street and highway |
| X958 | Assault by other and unspecified firearm discharge: other specified place |
| X959 | Assault by other and unspecified firearm discharge: unspecified place |
| X990 | Assault by sharp object: home |
| X999 | Assault by sharp object: unspecified place |
| Y009 | Assault by blunt object: unspecified place |
| Y094 | Assault by unspecified means: street and highway |
| Y099 | Assault by unspecified means: unspecified place |
| Y200 | Hanging, strangulation and suffocation, undetermined intent: home |
| Y240 | Other and unspecified firearm discharge, undetermined intent: home |
| Y289 | Contact with sharp object, undetermined intent: unspecified place |
| Y299 | Contact with blunt object, undetermined intent: unspecified place |
| Y350 | Legal intervention involving firearm discharge |
| **Suicide** | |
| X699 | Intentional self-poisoning by and exposure to other and unspecified chemicals and noxious substances: unspecified place |
| X700 | Intentional self-harm by hanging, strangulation and suffocation: home |
| X709 | Intentional self-harm by hanging, strangulation and suffocation: unspecified place |
| X720 | Intentional self-harm by handgun discharge: home |
| X748 | Intentional self-harm by other and unspecified firearm discharge: other specified place |
| **Drug/alcohol** | |
| F101 | Mental and behavioural disorders due to use of alcohol: harmful use |
| F102 | Mental and behavioural disorders due to use of alcohol: dependence syndrome |
| **Neurological disease** | |
| G931 | Anoxic brain damage, not elsewhere classified |
| G122 | Motor neuron disease |
| G309 | Alzheimer's disease, unspecified |
| I609 | Subarachnoid hemorrhage, unspecified |
| I619 | Intracerebral hemorrhage, unspecified |
| I639 | Cerebral infarction, unspecified |
| I64 | Stroke, not specified as hemorrhage or infarction |
| I694 | Sequelae of stroke, not specified as hemorrhage or infarction |
| **Infection** | |
| A09 | Infectious gastroenteritis and colitis, unspecified |
| A162 | Tuberculosis of lung, without mention of bacteriological or histological confirmation |
| A169 | Respiratory tuberculosis unspecified, without mention of bacteriological or histological confirmation |
| A419 | Sepsis, unspecified |
| B207 | HIV disease resulting in multiple infections |
| G009 | Bacterial meningitis, unspecified |
| I330 | Acute and subacute infective endocarditis |
| I38 | Endocarditis, valve unspecified |
| J180 | Bronchopneumonia, unspecified |
| J181 | Lobar pneumonia, unspecified |
| J189 | Pneumonia, unspecified |
| J440 | Chronic obstructive pulmonary disease with acute lower respiratory infection |
| K650 | Acute peritonitis |
| K658 | Other peritonitis |
| L031 | Cellulitis of other parts of limb |
| N390 | Urinary tract infection, site not specified |
| **Renal disease** | |
| I120 | Hypertensive renal disease with renal failure |
| N012 | Rapidly progressive nephritic syndrome with diffuse membranous glomerulonephritis |
| N179 | Acute renal failure, unspecified |
| N189 | Chronic renal failure, unspecified |
| N201 | Calculus of ureter |
| **Diabetes Mellitus** | |
| E140 | Unspecified diabetes mellitus: with coma |
| E141 | Unspecified diabetes mellitus: with ketoacidosis |
| E149 | Unspecified diabetes mellitus: without complications |
| **Other** | |
| D684 | Acquired coagulation factor deficiency |
| E668 | Other obesity |
| E669 | Obesity, unspecified |
| F329 | Depressive episode, unspecified |
| K254 | Gastric ulcer: chronic or unspecified with hemorrhage |
| K259 | Gastric ulcer: unspecified as acute or chronic, without hemorrhage or perforation |
| K290 | Acute hemorrhagic gastritis |
| K461 | Unspecified abdominal hernia with gangrene |
| K509 | Crohn's disease, unspecified |
| K550 | Acute vascular disorders of intestine |
| K559 | Vascular disorder of intestine, unspecified |
| K560 | Paralytic ileus |
| K574 | Diverticular disease of both small and large intestine with perforation and abscess |
| K578 | Diverticular disease of intestine, part unspecified, with perforation and abscess |
| K850 | Idiopathic acute pancreatitis |
| K859 | Acute pancreatitis, unspecified |
| K920 | Hematemesis |
| R98 | Unattended death |
| R99 | Other ill-defined and unspecified causes of mortality |
| Y340 | Unspecified event, undetermined intent: home |
| Y344 | Unspecified event, undetermined intent: street and highway |
| Y349 | Unspecified event, undetermined intent: unspecified place |
