# Supplementary material for: Impact on mortality of being seropositive for hepatitis C virus antibodies among blood donors in Brazil: A twenty-year study
Source: PLoS One. 2019 Dec 19;14(12):e0226566. doi: 10.1371/journal.pone.0226566 (PMC6922443; doi:10.1371/journal.pone.0226566)
Supplement: S2 Table — (DOCX) [file pone.0226566.s002.docx]

**S2 Table. Deaths organized by cause of death and by HCV serological status (2000-2016).**

| **Cause of Death** | **Number of Deaths** | | **Mortality rate**  **100,000 PY^***^ (IC95%)** | | **Crude HR^#^**  **(IC95%)**  **p-Value** | **Adjusted^&^ HR^#^**  **(IC95%)**  **p-Value** |
| --- | --- | --- | --- | --- | --- | --- |
|  | **Sero+***  **N=1,175** | **Sero-****  **N=2,528** | **Sero+**  16,002.60 PY | **Sero-**  34,882.40 PY |  |  |
| Deaths due to all causes | 74 | 64 | 462.43  (368.21-580.75) | 183.47  (143.61-234.41) | 2.51  (1.79–3.50)  <0.001 | 2.15  (1.53–3.02)  <0.001 |
| Deaths by causes associated with or directly related to HCV | 28 | 3 | 174.97  (120.81-253.41) | 8.6  (2.80-26.70) | 20.37  (6.19–67.01)  <0.001 | 16.28  (4.93 – 53.79)  <0.001 |

^*^Sero+: HCV seropositive only ^***^PY: Person-year

^**^Sero-: Seronegative for all infections tested ^#^HR: HazardRatio

^&^HR adjusted for sex, age, type of donation and type of donor
